# Dependence of intrafraction motion on fraction duration for pediatric patients with brain tumors

**DOI:** 10.1120/jacmp.v12i4.3609

**Published:** 2011-11-15

**Authors:** Chris Beltran, Thomas E. Merchant

**Affiliations:** ^1^ Department of Radiological Sciences St. Jude Children's Research Hospital Memphis TN

**Keywords:** pediatric, intrafraction motion

## Abstract

The purpose of this study was to quantify the intrafraction motion of pediatric patients with brain tumors during radiation therapy and investigate any correlation between motion, use of general anesthesia, and daily treatment duration. 100 pediatric patients with a mean age of 8.5 years (range: 1.0 to 17.8) were included in this prospective study. Forty‐one patients required general anesthesia during treatment, mean age 4.8 years; 59 patients did not, mean age 11.2 years. Each patient had an intracranial tumor and was treated in the supine position with a thermoplastic facemask and headrest for immobilization. A pretreatment localization CBCT was acquired for each treatment fraction and a post‐treatment CBCT was acquired every other fraction. If the magnitude of the patient's position pre‐CBCT offset was ≥2 mm, the position was corrected. The difference between the patient's position based on the post‐CBCT and the assumed position at the start of treatment (either the pre‐CBCT offset if the magnitude was < 2 mm, or 0 offset due to correction) was determined and labeled intrafraction motion. Correlations between daily treatment duration and intrafraction motion were examined. There was an average of 14.2 post‐CBCTs acquired per patient. The magnitude of the mean intrafraction motion was 1.2±0.8 mm for patients requiring general anesthesia, and 1.5±1.2 mm for those without (p<0.001). The mean offset in each direction was less than 0.5 mm for both cohorts. There was no correlation between daily treatment duration and the magnitude of intrafraction motion. The intrafraction motion of pediatric patients undergoing external beam therapy for intracranial tumors is small, < 2 mm, and is independent of the daily treatment duration.

PACS number: 87.53.Jw

## I. INTRODUCTION

Recently, we reported results of a clinical trial using daily cone‐beam CT (CBCT) to evaluate setup uncertainty in pediatric patients with brain tumors.^(^ ^1^ ^)^ Based on the data from 100 children, we found that the setup uncertainty could be limited to 2 mm. We noted that there was some dependence on patient positioning (supine vs. prone) and the use of general anesthesia (yes vs. no). We found that children who were not localized using CBCT had a setup uncertainty of 4 mm. In that study, we also showed that the intrafractional uncertainty was approximately 2 mm; however, we did not correlate this motion with treatment fraction duration.

In the adult population, Hoogeman et al.^(^ ^2^ ^)^ found that intrafraction motion increases linearly with time for both intra‐ and extracranial patients. A concern that has not been addressed as of yet is whether or not treatment duration has an impact on intrafraction motion for pediatric patients. This has become a concern in our clinic as we are currently using a daily image‐guided protocol for all patients and increasing the use of IMRT, both of which increase fraction duration. The purpose of this study is to quantify the relation between intrafractional motion and treatment fraction duration for pediatric patients with brain tumors.

## II. MATERIALS AND METHODS

One hundred pediatric patients enrolled on a prospective daily localization protocol^(^ ^1^ ^)^ were included in this study. Criteria for inclusion included age < 18 years, intracranial target, >20 treatment fraction with daily localization, and treated in the supine position. The mean age of the included cohort was 8.5 years (range: 1.0 to 17.8), and 51 were female. Forty‐one patients required general anesthesia during treatment, mean age 4.8 years; 59 patients did not, mean age 11.2 years.

Each patient was immobilized with a thermoplastic facemask and headrest. Regarding the localization protocol, a pretreatment localization CBCT was acquired for each treatment fraction and a post‐treatment CBCT was acquired every other fraction. The CBCT used was an investigational low dose MV device which delivers 1 cGy per CBCT at isocenter.^(^ ^3^ ^,^ ^4^ ^)^ If the magnitude of the patient's pre‐CBCT offset was ≥2 mm, the position was corrected. The difference between the patient's position (lateral, longitudinal, and vertical) based on the post‐CBCT and the assumed position at the start of treatment was determined and labeled intrafraction motion. The assumed position was either the pre‐CBCT offset if the magnitude was < 2 mm or 0 if there was corrective action taken. Correlations between fraction duration and intrafraction motion were examined for patients treated with and without general anesthesia. Fraction duration was taken to be the elapsed time from the start of the acquisition of the pre‐CBCT to the start of acquisition of the post‐CBCT as recorded from our record and verify system.

## III. RESULTS

For the 100 patients, 1423 fractions had both pre‐ and post‐CBCTs. That gives an average of 14.2±1.5 fractions per patient that were evaluated in this study. The mean fraction duration was 14.6±4.1 minutes. For all patients the mean lateral, longitudinal and vertical offsets were 0.0±0.9, −0.2±1.0, and 0.2±1.1 mm, respectively. The mean magnitude was 1.3±1.1 mm. Table 1 summarizes these values for patients with and without general anesthesia. Examining the magnitude, the difference in the two cohorts is statistically significant (p‐value < 0.001); however, clinically the difference is small — offset mean magnitude of 1.2 mm for the cohort which received general anesthesia vs. 1.5 mm for those without.

**Table 1:** The mean and standard deviation (std‐dev) values for the number of fractions, fraction duration in minutes (min), lateral (Lat), longitudinal (Long), vertical (Vert), and magnitude in mm are shown for all patients — the cohort treated with general anesthesia (GA) and the cohort treated without (No GA).

		*Fractions*	*Duration*	*Lat*	*Long*	*Vert*	*Magnitude*
All	mean	14.2	14.6 min	0.0 mm	−0.2 mm	0.2 mm	1.3 mm
(n=100)	std‐dev	1.5	4.1	0.9	1.0	1.1	1.1
GA	mean	14.2	14.5	−0.1	−0.2	0.1	1.2
(n=41)	std‐dev	1.8	4.5	0.8	0.8	0.8	0.8
No GA	mean	14.3	14.7	0.0	−0.3	0.3	1.5
(n=59)	std‐dev	1.3	3.9	0.9	1.0	1.1	1.1

**Figure 1:**
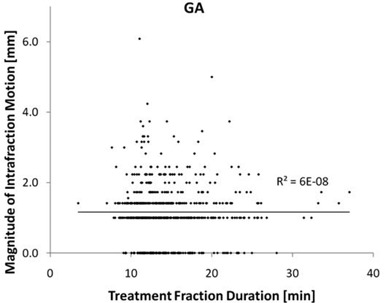
Scatter plot of the fraction duration vs. the magnitude of intrafraction motion for the cohort treated with general anesthesia (GA). The trend line is shown with the Person $R^{2}$ value.

**Figure 2:**
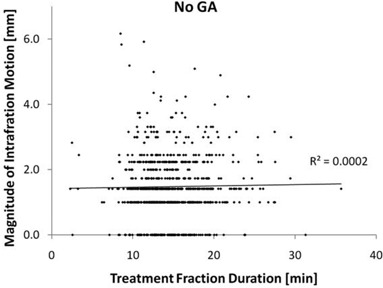
Scatter plot of the fraction duration vs. the magnitude of intrafraction motion for the cohort treated without general anesthesia (No GA). The trend line is shown with the Person $R^{2}$ value.

There was no correlation found between fraction duration and intrafraction motion for either cohort. Figures 1 and 2 display scatter plots of the fraction duration vs. intrafraction offset magnitude of both cohorts. The Pearson $R^{2}$ value is close to zero for both cohorts. In addition, dividing the general anesthesia cohort into two groups by fraction duration gives a mean duration of 11.3 minutes for the short duration group and 17.7 for the long duration group. The mean magnitude offset is 1.2±0.9 mm for the short group and 1.2±0.8 mm for the long group (p=0.65). Further dividing this cohort into the 50 with the shortest and with the longest fraction duration gives a mean time of 9.0 and 25.0 minutes, respectively. The mean magnitude for the short duration group is 1.2±0.6 mm and 1.2±0.8 for the long duration group (p=0.74). A similar result is found for the cohort without anesthesia.

## IV. DISCUSSION & CONCLUSION

In this report, we have shown that the interfraction motion for pediatric patients with brain tumors treated in the supine position is small, with magnitude and standard deviations of less than 2 mm. This is similar to our previous findings,^(^ ^1^ ^)^ and the findings in the adult population for intracranial tumors.^(^ ^2^ ^,^ ^5^ ^,^ ^6^ ^)^ However, unlike Hoogeman et al.^(^ ^2^ ^)^ who found that the amount of intrafraction motion increased with fraction duration, we found no correlation between fraction duration and intrafraction motion. This is a key finding that is based on a large cohort with many observations. Therefore, while minimal treatment time for patient comfort and anesthesia concerns is still important, the duration of treatment should not be a concern when determining treatment margins for pediatric patients treated in the supine position for intracranial targets. The treatment position and site is critical, as this study did not investigate prone patients or patients with extracranial targets.

## ACKNOWLEDGMENTS

This work was supported in part by Siemens Medical Systems and the American Lebanese Syrian Associated Charities (ALSAC).
